# Exploring the abomasal lymph node transcriptome for genes associated with resistance to the sheep nematode *Teladorsagia circumcincta*

**DOI:** 10.1186/1297-9716-44-68

**Published:** 2013-08-08

**Authors:** Anton Gossner, Hazel Wilkie, Anagha Joshi, John Hopkins

**Affiliations:** 1 The Roslin Institute & R(D)SVS, University of Edinburgh, Easter Bush, Midlothian EH25 9RG, UK

## Abstract

This study exploited Blackface lambs that varied in their resistance to the abomasal nematode parasite, *Teladorsagia circumcincta*. Infection of these lambs over 3 months identified susceptible (high adult worm count, high faecal egg count and low IgA antibody) and resistant animals that had excluded all parasites. Previous work had shown that susceptibility and resistance is dependent on the differential immune response to the parasite, which occurs within the abomasal (gastric) lymph node (ALN) that drains the site of infection. The Affymetrix ovine gene array was used to interrogate the transcriptome of the ALN to identify genes and physiological pathways associated with resistance. We used a bovine RT-qPCR array of 84 genes to validate the gene array, and also report digital gene expression analysis on the same tissues, reanalysed using the Oar v3.1 sheep genome assembly. These analyses identified Humoral Immune Response, Protein Synthesis, Inflammatory Response and Hematological System Development and Function as the two top-ranked networks associated with resistance. Central genes within these networks were *IL4*, *IL5*, *IL13RA2* and in particular *IL13*, which confirmed that differential activation of Th2 polarized responses is critical to the resistance phenotype. Furthermore, in resistant sheep there was up-regulation of genes linked to control and suppression of inflammation. The identity of differentially-expressed chemokines and receptors in the resistant and susceptible sheep also begins to explain the cellular nature of the host response to infection. This work will greatly help in the identification of candidate genes as potential selectable markers of genetic resistance.

## Introduction

Gastrointestinal nematode parasites are the cause of major economic losses to the sheep agricultural industry [1] and the major species in cool temperate regions is the abomasal strongylid *Teladorsagia circumcincta*[1,2]. The control of this parasite is largely by the use of broad-spectrum anthelmintics [3,4] but the increasing incidence of drug-resistant parasites and concern of drug residues in meat [5] has led to the search for alternative methods of parasite management [6]. The animals most susceptible to *T. circumcincta* are weaned lambs [7]. Most lambs eventually suppress infection [8] through the development of IgE and IgA anti-parasite antibodies; but this takes more than 6 weeks of persistent infection with infectious larvae [9-11]. Mucosal mast cells have also been shown to play an important role in the limitation of larval colonization and expulsion of helminths [12,13] and these also function largely in association with parasite-specific antibodies [14]. However, some sheep in most flocks develop only low levels of helminth-specific antibodies and fail to control larval colonization and egg production. Indeed, IgA levels and faecal egg counts (FEC) have been used as selectable markers for resistance [8,15,16]. Furthermore, different sheep breeds show marked diversity in resistance to helminth infection [17-19]. Consequently, one strategy for the non-pharmacological control of parasites is the exploitation of genetic variation for resistance found within and between different sheep breeds [20,21].

Selection for resistance can be based on quantitative measurements of one or more phenotypic traits such as FEC and IgA antibody levels [16,22] but the identification of molecular markers is potentially a more reliable approach for high resolution selection [23]. There are three approaches for the identification of such markers, quantitative trait locus (QTL) mapping, genome-wide association studies (GWAS) and candidate gene analysis [24]. QTL mapping is of low power and requires extensive further work to identify candidate genes [25]. GWAS is expensive, requiring very large numbers of samples; in addition, lack of current sheep genomic resources mitigates against high resolution analysis [26]. The alternative candidate gene approach aims to evaluate the relationship between a phenotypic trait and a variation in a gene; this gene is selected by measuring differential expression in relation to a relevant phenotype. A number of studies have used sheep microarrays to identify genes and molecular pathways associated with host responses to abomasal nematode parasites in sheep. Most have analysed the transcriptome of the *Haemonchus contortus* or *T. circumcincta* infected abomasal mucosa [27-29] or afferent lymph cells draining that mucosa [30]. In addition, a RT-qPCR assay has been developed to analyse a limited number of immune-inflammatory genes [31]. However, the immune response to parasites in the abomasum takes place within the abomasal (gastric) lymph node (ALN) and the events within that node determine the quality and quantity of the immune response and consequently the clinical outcome of infection.

This current study exploited parasite-naïve Blackface lambs with diversity in their predicted genetic resistance to *T. circumcincta*, which were trickle-infected with L3 larvae to mimic natural infection [32]. This regime resulted in lambs with a range of resistance as assessed by adult worm counts, FEC and IgA levels. Previous studies with these sheep used digital gene expression (DGE) [24] and RT-qPCR [33] to conclude that both resistance (no FEC/high IgA) and susceptibility (high FEC/low IgA) are active responses to infection; and that the inflammatory lesions of the susceptible sheep are associated with differential activation of Th17 T cells. Consequently the aim of this project was to investigate genes and physiological pathways associated with the differential activation of the immune response linked to the different disease outcomes. These pathways are likely to contain candidate genes as potential selectable markers for resistance to *T. circumcincta* infection. However, in this new study we use the novel Affymetrix Ovine Gene 1.1 ST whole-genome array, based on the homologous Oar v2.0 assembly, and focus on gene and pathway identity in relation to resistance and susceptibility.

## Materials and methods

### Animals and experimental design

Fifty-five female Blackface lambs (10–13 weeks old), from a flock previously used for quantitative genetic and QTL analyses [23], were housed in worm-free conditions. Ten lambs were sham infected controls; 45 lambs were infected experimentally with ~2300 infective L3 *T. circumcincta* larvae three times a week for 12 weeks and sacrificed two days after the last infection. The sham-infected controls (C) were twins of lambs in the infected group. At *post mortem* ten infected lambs had no detectable adult worms in the total abomasal contents, while the other infected lambs had a range of adult worm counts up to 11 300. The lambs selected for analysis were chosen to maximize the power of detecting differential expression. Consequently, animals were ranked according to their infection levels [32]. The 7 most resistant lambs (R) had no detectable abomasal adult worms or faecal egg count (FEC), high IgA antibody levels and high body weight. The 7 most susceptible lambs (S) were those with the highest adult worm count (mean 6000, maximum 11 300), high FEC (mean 414, maximum 950), low IgA antibody levels and low body weight. Details of the animals, infection protocols, trait and population genetic analyses have been described previously [32,33]. Animal experiments were approved by University of Edinburgh Ethical Review Committee and conducted under an Animals (Scientific Procedures) Act 1986 Project Licence.

### Sample collection and total RNA isolation

Abomasal (gastric) lymph nodes (ALN) were removed immediately post mortem and stored at −80 °C in RNAlater (Ambion, Huntingdon, UK). Total RNA was isolated using the Ribopure Kit (Ambion) as described previously [32]. RNA quality and integrity were assessed using a RNA 6000 Nano LabChip on the Agilent 2100 Bioanalyzer and quantified using a NanoDrop ND-1000 spectrophotometer; all had an RNA Integrity Number of > 7.5.

### Whole-transcript expression analysis and profiling

Primary transcriptome analysis was by Affymetrix Ovine Gene 1.1 ST Array. Sense-strand cDNA was generated from total RNA (500 ng) subjected to two rounds of amplification (Ambion® WT Expression Kit). The obtained cDNA was used for biotin labelling and fragmentation by Affymetrix GeneChip® WT Terminal Labelling and Hybridization kit (Affymetrix). Biotin-labelled fragments of cDNA (5.5 μg) were hybridized to Affymetrix Ovine Gene 1.1 ST Array plates using the appropriate Hyb-Wash-Scan protocol for this plate and the Gene Titan Hyb Wash Stain kit for the reagents (Affymetrix). After hybridization every array plate was washed and stained before the array plates were scanned by the Imaging Station of GeneTitan System. Image generation and the resulting CEL files for analysis were produced in Affymetrix® GeneChip® Command Console® Software (AGCC) version 3.0.1. Initial QCs were performed in Expression Console. The obtained Affymetrix .CEL files were imported into the Genomics Suite software package version 6.13.0213 (Partek, St. Louis, MO, USA). The imported data were analysed at the gene-level, with exons summarized to genes, using the mean expression of all the exons of a gene. Background correction was carried out using the robust multiarray average (RMA) algorithm, with quantile normalization, median polish probe summarization, and log2 probe transformation. Differentially expressed genes were identified by ANOVA, genes with a fold change > 1.5 or < −1.5, and a false discovery rate (FDR) > 0.05 were kept, calculated using the Benjamini–Hochberg method to adjust *P*-values [34]. Gene annotation was performed based on similarity scores in BLASTN comparisons against ovine or bovine sequences in GenBank.

### Inflammatory cytokines & receptors RT2 profiler™ PCR array

Quantitative real-time RT-PCR (RT-qPCR) analysis was performed using the SABiosciences Cow Inflammatory Cytokines & Receptors RT2 Profiler™ PCR Array (Qiagen, Crawley, UK), which measures the expression of 84 genes that mediate the inflammatory response (Cat. no. 330231 PABT-011ZR). Total RNA was extracted as described above and treated with RNase-free DNase I (Qiagen) and RNeasy MinElute Cleanup Kit (Qiagen) according to the manufacturer’s protocol to eliminate DNA contamination. Each 0.8 μg sample of RNA was reverse transcribed using a RT^2^ First Strand Kit (Qiagen) before dilution with RNase-free water according to the manufacturer’s protocol. A real time PCR was performed on each cDNA sample using the RT^2^ Profiler™ PCR Array with the RT^2^ SYBR Green ROX FAST Mastermix on a Rotor-Gene Q cycler (Qiagen). The cycling profile was performed at 95 °C for 10 min, followed by 40 cycles of 95 °C for 15 s and 60 °C for 1 min. Melting curve analysis of PCR products confirmed the absence of secondary product. RT^2^ Profiler PCR Array Data Analysis v3.5 was used for data analysis. The data analysis was based on the ΔΔCt method with gene expression normalized to the reference gene *YWHAZ*.

### Cloning of ovine gene fragments

Amplicons from the RT^2^ Profiler™ PCR Array were cloned and sequenced to confirm PCR primer specificity using the TOPO® TA Cloning® Kit for Sequencing (Life Technologies) and BigDye® Terminator v3.1 Cycle Sequencing Kit (Applied Biosystems) according to the manufacturer’s instructions. Cloned amplicon sequences were used in BLASTN comparisons against ovine or bovine sequences in GenBank to confirm identity.

### Illumina digital gene expression

Full details of the RNAseq methods and analysis have been described previously [24]. These sheep sequences were originally aligned against the Btau 4.0 bovine genome. Detailed protocols, metadata and all raw data are deposited at the ArrayExpress database [35] accession number E-MTAB-445. For the current study the raw data were reanalysed by alignment against the sheep genome assembly Oar v3.1 [36] using Bowtie v0.12.8 [37]. Only tags with phred mapping quality of at least 30, with a maximum of one base-pair mismatch and mapped to less than two genome locations were retained for further analysis. Furthermore, only genes that were mapped from five samples or more were included in the final analysis. Statistical analysis was performed using “R v2.15”, and Limma [38] within BioConductor 2.11 [39] was used to calculate the differential gene expression between resistant (R), susceptible (S) and control (C) groups, including fold change and q value, which is analogous to an adjusted p value or false discovery rate (FDR). Only genes with a fold change greater than 1.5 and q value ≤ 0.05 (FDR 5%) were annotated.

### Molecular network and pathway analysis

The Ingenuity Pathways Analysis (IPA) Spring Release (2013) Software (Qiagen) was used to identify networks of interacting genes and other functional groups from the datasets of differentially-expressed genes. DEG was analysed, by uploading the HUGO Gene Nomenclature Committee (HGNC) gene symbols for the sheep orthologues and fold change data to IPA.

## Results

### Expression analysis by Affymetrix ovine gene array

All microarray data, metadata and protocols are available in the ArrayExpress database under accession number E-MTAB-1580. The Affymetrix Ovine Gene 1.1 ST Arrays identified 43 genes (Table 1) in the ALN that showed significant difference (fold change ≥ 1.5 and adjusted *p* value ≤ 0.05) in the R vs. C comparison, four genes in the S vs. C comparison and only three genes in the R vs. S comparison. All differentially-expressed genes in the S vs. C and R vs. S comparisons were also significantly differentially-expressed in the R vs. C comparison except *VIRP2* that was 2.04 fold increased in susceptible sheep compared to controls.

**Table 1:** Differentially-expressed genes in ALN as assessed by Affymetrix ovine gene array.

			**R vs.S**		**R vs. C**		**S vs. C**	
**Transcript**	**Accession number**	**Gene**	*p*-value*	**FC**^**§**^	*p*-value*	**FC**	*p*-value*	**FC**
14836730	NM_001082594	IL13	**7.44E-07**	**3.17**	**1.87E-08**	**4.76**	0.0195	1.50
14863257	XM_004003413	COL6A5	**8.64E-07**	**8.69**	**4.88E-07**	**10.47**	0.5421	1.21
14722918	XM_004005214	CTNNAL1	2.52E-06	1.45	**4.03E-07**	**1.56**	0.2107	1.08
14726591	XM_004003604	CHI3L2	0.1340	−1.15	**8.18E-06**	**1.82**	**5.15E-07**	**2.10**
14830831	XM_004018378	IL17RB	0.0061	1.78	**3.13E-07**	**4.73**	9.17E-05	2.66
14710165	M84356	IGHE	0.4861	1.30	**9.63E-07**	**17.21**	**3.35E-06**	**13.25**
14793854	XM_004020976	CCL26	3.37E-05	6.55	**5.08E-07**	**15.50**	0.0252	2.37
14806387	XM_004006081	ACTG2	**2.09E-06**	**3.12**	**6.66E-06**	**2.94**	0.7353	−1.06
14795035	XM_004018317	IL5RA	0.0031	1.64	**6.59E-07**	**3.15**	0.0004	1.92
14860591	XM_004008559	FCER2	0.3480	−1.09	**1.08E-05**	**1.72**	**1.88E-06**	**1.87**
14724715	NM_001142892	NFIL3	0.0241	1.21	**1.76E-06**	**1.77**	0.0002	1.46
14842210	AC150860	unknown	0.0002	1.95	**3.49E-06**	**2.69**	0.0422	1.38
14731097	XM_004021707	PDLIM3	0.0420	1.19	**3.46E-06**	**1.74**	0.0002	1.46
14780240	XM_004007330	IL1RL1	0.0001	2.09	**5.90E-06**	**2.70**	0.1112	1.29
14763982	XM_004015182	FFAR2	0.0002	1.70	**6.95E-06**	**2.09**	0.0921	1.23
14894439	NM_001009749	SELE	0.4637	1.09	**4.34E-05**	**−1.88**	1.03E-05	−2.05
14776999	XR_083707	EMR3	0.0006	4.02	**6.48E-06**	**9.29**	0.0278	2.31
14767454	XM_004011302	CD73	0.6156	1.04	**1.76E-05**	**1.65**	4.74E-05	1.58
14814624	XM_004021547	ANXA8	0.0470	1.47	**7.46E-06**	**3.25**	0.0005	2.22
14855554	XM_004018137	ASB2	0.1414	1.14	**1.14E-05**	**1.72**	0.0002	1.51
14826002	XM_004012071	CYSLTR2	0.0002	1.42	**2.31E-05**	**1.56**	0.2324	1.10
14826670	XM_004003026	APOD	0.0074	1.75	**1.19E-05**	**3.21**	0.0056	1.83
14872046	XM_004012573	P2RX1	9.97E-05	1.88	**6.98E-05**	**1.97**	0.7164	1.05
14724584	XM_004022410	IL13RA2	0.0019	2.34	**1.92E-05**	**4.10**	0.0331	1.75
14873273	XM_004002636	CD1A	0.0455	1.31	**1.92E-05**	**2.15**	0.0015	1.64
14722365	XM_004015563	CCL17	0.5259	1.15	**4.54E-05**	**3.33**	0.0002	2.90
14766308	XM_004002922	CD200R1	0.0134	2.48	**1.99E-05**	**7.36**	0.0054	2.97
14789329	XM_004004106	SLC28A3	0.0006	2.33	**3.76E-05**	**3.15**	0.1660	1.35
14894373	NM_001009251	LGALS14	0.0166	2.44	**2.72E-05**	**7.25**	0.0061	2.98
14789001	XM_004023684	SMPD3	0.0002	2.17	**0.0001**	**2.30**	0.7191	1.06
14854660	XM_004018529	HRH1	0.0032	1.31	**3.40E-05**	**1.57**	0.0365	1.20
14724104	XR_173262	ALOX15	0.0007	5.81	**5.48E-05**	**10.68**	0.1877	1.84
14768669	XM_004013559	SLC45A3	0.0003	1.50	**9.12E-05**	**1.61**	0.4658	1.07
14710170	AF024645	IGHA	0.0023	1.55	**4.03E-05**	**2.01**	0.0559	1.30
14809379	XM_004003929	COL6A2	0.0059	1.43	**3.89E-05**	**1.93**	0.0237	1.34
14773724	XM_004022514	CRLF2	0.0015	1.66	**9.79E-05**	**2.03**	0.1733	1.22
14894389	NM_001009425	CD1B	0.0031	1.54	**9.02E-05**	**1.94**	0.0921	1.26
14828762	NM_181018	CLCA3	0.0082	1.64	**7.66E-05**	**2.43**	0.0346	1.48
14713579	AC225835	CCL3L3	0.0391	1.23	**7.62E-05**	**1.65**	0.0076	1.34
14864484	XM_004022897	CD1B	0.0091	1.39	**9.87E-05**	**1.79**	0.0401	1.29
14710851	XM_004006654	NR1H4	0.0267	1.21	**0.0001**	**1.51**	0.0153	1.25
14891746	XM_004012107	TRPC4	0.0128	1.35	**0.0001**	**1.75**	0.0324	1.30
14784733	XM_004015344	CABP5	0.0183	−1.25	**0.0001**	**−1.56**	0.0235	−1.25
14732131	XM_004008376	VIPR2	0.0420	−1.28	0.0010	1.59	**1.27E-05**	**2.04**

*P value with no FDR applied. ^§^FC; fold change. Bold; adjusted *p* ≤ 0.05 after 5% FDA.

**Figure 1:**
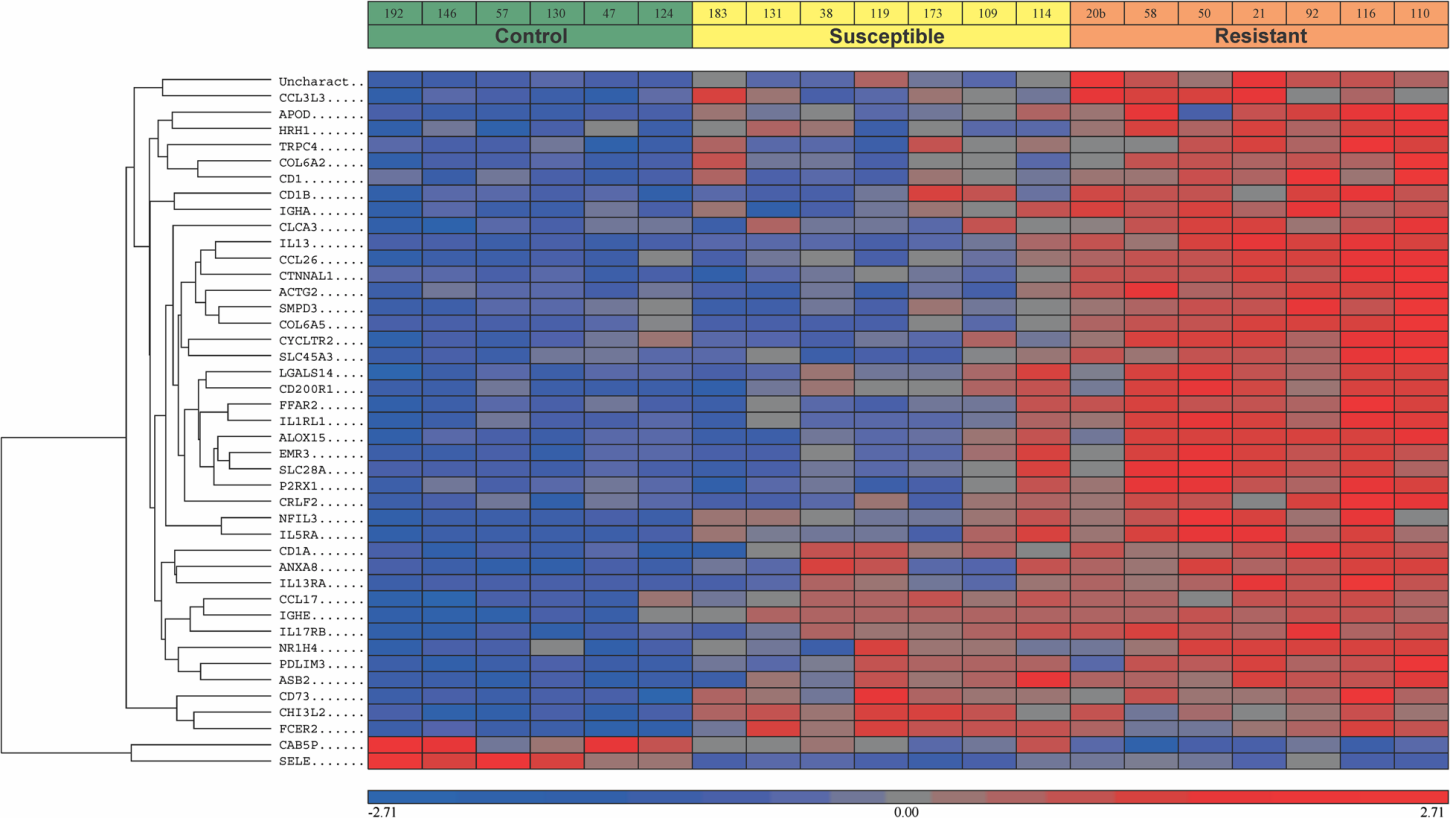
**Heat map of differentially-expressed genes.** Data from Affymetrix ovine gene array analysis showing changes in all genes identified as ≥ 1.5 fold and adjusted *p* ≤ 0.05, in the three comparisons R vs. C, S vs. C and R vs. S (Table 1). Each row represents a gene. The relative levels of expression are represented by the intensity of colour; red, increased expression and blue decreased expression within each comparison.

Presentation of these data by heat map (Figure 1) illustrates that the overwhelming majority of the differentially-expressed genes are increased in the infected groups. Forty-one of the 43 genes in the R vs. C comparison are increased in the R group; all four of the genes in the S vs. C comparison were increased in the S group and all three in the R vs. S comparison were increased in the R group. *IL13*, *COL6A5* and *ACTG2* were significantly increased in the R animals when compared to both the S and C groups. *CHI3L2*, *IGHE* and *FCER2* were significantly increased in both the R vs. C and S vs. C comparisons but not in the R vs. S comparison. *VIPR2* was the only gene significantly increased in the S animals and not in the R group when compared to uninfected controls.

### RT-qPCR array validation

Validation of the Affymetrix gene arrays was performed using the SABiosciences Cow Inflammatory Cytokines & Receptors RT^2^ Profiler™ PCR Array which consists of 84 key genes that mediate inflammation. This assay is optimized for bovine genes and data were obtained for 83 genes when used with sheep cDNA (Additional file 1). The mean sequence identity between these 83 bovine and ovine genes is 96% (minimum 91%, IL27; maximum 100%, TNFSF13B). Single distinct melt curves obtained for all genes, except for *NAMPT* (no data), in all samples confirmed the specificity of the assay. Furthermore, analysis of the sequences of ten sheep amplicons selected randomly (*CCL11*, *CXCL13*, *IL2RB*, *IL2RG*, *IL4*, *IL6R*, *IL13*, *IL16*, *PF4*, *TNFSF4*) and produced using the assay primers, showed them to be 100% identical to sequences of the respective sheep homologues.

Table 2 identifies those genes showing ≥ 1.5 fold differential expression in the three comparisons. Three genes were significantly differentially expressed (*p* ≤ 0.05) in the R vs. C comparison, the Th2 cytokines *IL4* and *IL13* were increased by 11.9 and 3.96 fold respectively and *MIP* was repressed by 2.16 fold, in resistant sheep. *IL4* (3.83 fold) and *IL13* (3.37 fold) were also significantly increased, and *CCL5* (2.43 fold), *CCL11* (2.11 fold), *CXCR1* (2.49 fold), *CXCR3* (2.25 fold) and *TNFSF10* (2.03 fold) were significantly repressed in the susceptible animals when compared to uninfected controls. In the comparison of the two infected groups the Th2 cytokines *IL5* (3.4 fold) and *IL13* (3.54 fold) were significantly increased and the chemokine *CCL5* (3.16 fold) was repressed in resistant sheep.

**Table 2:** Differential expression of inflammatory cytokines and receptors in ALN as assessed by RT-qPCR.

**Up-regulated genes**	Fold change*	***p*-value**	**Down-regulated genes**	**Fold change**	***p*-value**
Resistant vs. Control					
IL4	**3.96**	**0.006**	AIMP1	−3.26	0.980
IL5	2.86	0.098	BMP2	−2.67	0.914
IL10RA	2.43	0.063	CCL20	−2.57	0.119
IL13	**11.92**	**0.001**	CXCL12	−7.28	0.297
IL17B	2.06	0.104	IL2RB	−4.33	0.135
IL9R	4.01	0.254	MIF	**−2.16**	**0.020**
			TNFSF4	−3.42	0.112
			VEGFA	−4.28	0.208
Susceptible vs. Control					
IL4	**3.83**	**0.007**	CCL5	**−2.43**	**0.008**
IL13	**3.37**	**0.030**	CCL11	**−2.11**	**0.046**
			CXCL10	−2.76	0.182
			CXCL12	−2.24	0.263
			CXCL9	−3.00	0.106
			CXCR1	**−2.49**	**0.006**
			CXCR3	**−2.25**	**0.005**
			IFNG	−2.14	0.135
			IL2RB	−4.71	0.100
			TNFSF10	**−2.03**	**0.020**
Resistant vs. Susceptible					
CCL5	**3.16**	**0.030**	AIMP1	−2.90	0.773
CCR2	2.58	0.185	CXCL12	−3.26	0.823
CXCL9	2.92	0.165	TNFSF4	−2.13	0.424
CXCL10	3.07	0.099	VEGFA	−2.77	0.797
IFNG	2.16	0.129			
IL5	**3.40**	**0.045**			
IL13	**3.54**	**0.002**			
IL17B	2.71	0.061			
IL9R	3.77	0.200			

*Fold change is the ratio of normalized mean expression between resistant, susceptible and control groups. Bold is *p* ≤ 0.05.

**Figure 2:**
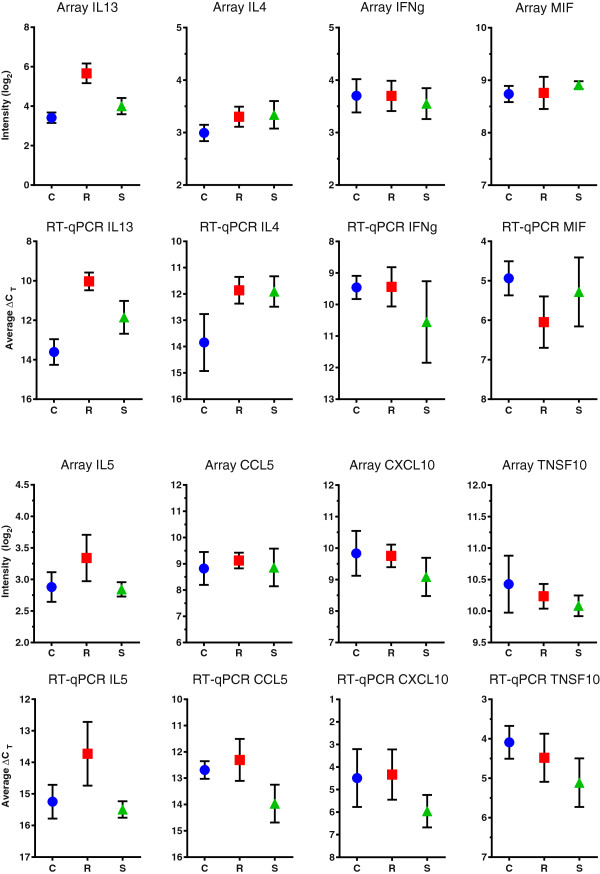
**Comparison of Affymetrix ovine gene and PCR arrays.** PCR array results shown are the average ΔCT for the gene of interest (GOI) calculated using the following formula ΔCT = C_T_^(GOI)^- C_T_^(YWHAZ)^ and error bars show the standard deviation. The microarray values plotted are the mean normalized log2 intensity values. Blue circles represent the control (C); red squares resistant (R) and green triangles susceptible (S) samples.

Spearman’s rank correlation analysis of the fold changes calculated for all 83 genes in the R vs. C and S vs. C comparisons (comparing 164 values) gave a low, but highly significant positive correlation (ρ = 0.37, *P* < 0.0001) between the data obtained by the Affymetrix and PCR arrays. A direct comparison of quantitative expression of eight selected genes (Figure 2) shows the close relationship between the relative levels of expression measured by the two independent methods.

### Ingenuity pathway analysis

**Figure 3:**
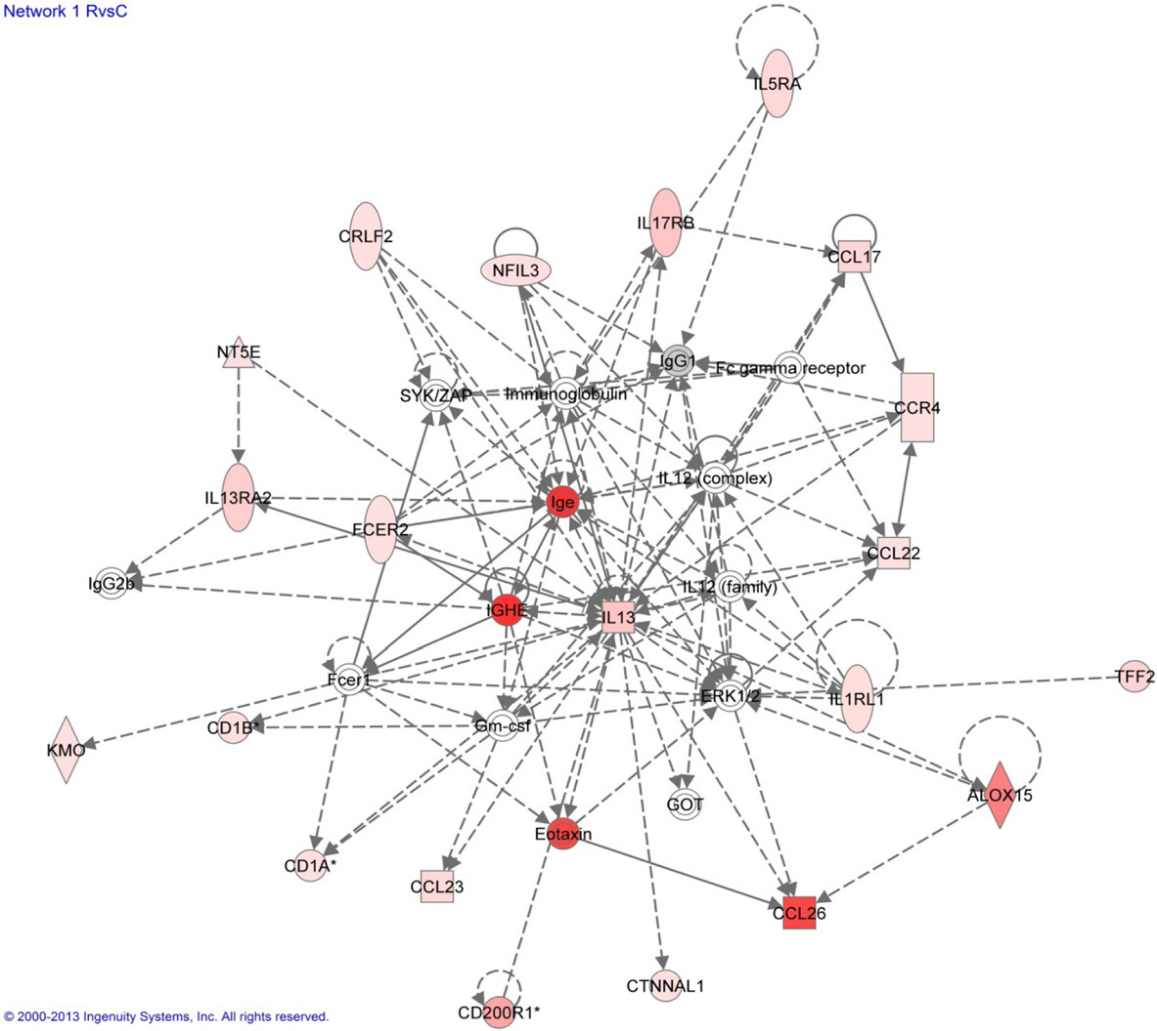
**Ingenuity pathway analysis of the top-ranked network.** The top- ranked network is “Humoral Immune Response, Protein Synthesis, Inflammatory Response” with a P-score of 48 based on Ingenuity Pathway Knowledge Base. Analysis used differentially-expressed genes from the Affymetrix Gene Array with ≥ 1.5 fold change, *p*-values of ≤ 0.05 and a FDR of 0.15. The shape of the nodes indicates the major function of the protein expressed by that gene. Red coloured nodes (genes) are up-regulated and the density relates to expression levels. Uncoloured nodes represent genes not identified as differentially-expressed in the current study. A solid line indicates direct evident and a dashed line indirect evidence of interaction. The arrow head show the direction of interaction.

IPA was used to help characterize how individual differentially-expressed genes interact, and consequently influence biological processes that affect the development of resistance or the maintenance of susceptibility to chronic *T. circumcincta* infection. Analysis of the data obtained using the Affymetrix gene array identified seven networks (Table 3), five with the R vs. C comparison and one each with the S vs. C and R vs. S comparisons. The top two ranked networks for the R vs. C dataset were Humoral Immune Response, Protein Synthesis, Inflammatory Response (Figure 3) and Hematological System Development and Function, with P-scores of 48 and 28 respectively. The three top Bio Functions, beyond the *p* ≤ 10^-10^ threshold, within these networks (Table 4a) were Immunological Disease with 24 genes with the highest *p* value of 2.73 × 10^-13^, Inflammatory Disease with 41 genes *p* = 8.34 × 10^-13^, and Hypersensitivity Response with 19 genes *p* = 7.98 × 10^-11^ associated with Diseases and Disorders and Hematological System Development and Function with 37 genes *p* = 4.79 × 10^-12^, Tissue Morphology with 33 genes *p* = 4.79 × 10^-12^, and Humoral Immune with 13 genes *p* = 2.28 × 10^-11^, associated with Physiological System Development and Function.

**Table 3:** Top networks identified by ingenuity pathway analysis, from the Affymetrix ovine gene array.

																																																																					**Resistant vs. Control**	**Score**
																																																																					Humoral Immune Response, Protein Synthesis, Inflammatory Response	48
																																																																					Haematological System Development and Function, Haematopoiesis, Tissue Morphology	28
																																																																					Cardiovascular System Development and Function, Cellular Movement, Gene Expression	22
																																																																					Hereditary Disorder, Skeletal and Muscular Disorders, Tissue Morphology	21
																																																																					Cell Signalling, Molecular Transport, Vitamin and Mineral Metabolism	20
																																																																					**Susceptible vs. Control**																																								
																																																																					Cell-To-Cell Signalling and Interaction, Haematological System Development and Function, Immune Cell Trafficking	24
																																																																					**Resistant vs. Susceptible**																																								
																																																																					Antigen Presentation, Lipid Metabolism, Small Molecule Biochemistry	18

**Table 4:** Top bio functions identified by ingenuity pathway analysis, from the Affymetrix ovine gene array.

**a. R vs. C comparison**	
**DISEASES AND DISORDERS**	***P*-value**
**Immunological Disease**	
CSF3R, SELE, PTGDR2, IL1RL1, IGHE, VIPR2, CCL23, NR1H4, IL13RA2, CCL17, CCL22, IL13, ADRA1D, HRH1, IL17RB, CCR4, CCL2, NLRP12, CD1A, CCL26, CFH,HRH4, CYSLTR2, FCER2.	2.73 × 10^-13^ - 6.71 × 10^-03^
**Inflammatory Response**	
GATA1, CRLF2, NR1H4, VIPR2, IL1RL1, IL13RA2, SOCS2, P2RX1, CCL17, SLC9A4, CCL22, HRH1, NFIL, TFF2, CCL2, CD1A, HPGD, CFH, CYSLTR2, FCER2, CSF3R, ALOX15, SELE, PTGDR2, IL5RA, CCL23, IGHE, CD200R1, CD1B, ULBP1, P2RX7, IL13, IL17RB, CCR4, NT5E, NLRP12, PILRA, CCL26, IGHA1, SH2D1B, HRH4.	8.34 × 10^-13^ - 6.71 × 10^-03^
**Hypersensitivity Response**	
ALOX15, PTGDR2, SELE, IL5RA, GATA1, VIPR2, IL1RL1, IGHE, CD200R1, CCL17, CCL22, P2RX7, IL13, IL17RB, CCR4, CCL2, CCL26, HRH4, FCER2	7.98 × 10^-11^ - 4.94 × 10^-03^
**Dermatological Diseases and Conditions**	
ALOX15, PTGDR2, SELE, IGHE, VIPR2, NR1H4, CCL23, CCL17, CCL22, IL13, ADRA1D, HRH1, IL17RB, CCL2, CCR4, NT5E, CD1A, HPGD, HRH4, CYSLTR2, FCER2	1.78 × 10^-09^ - 6.71 × 10^-03^
**Inflammatory Disease**	
CRLF2, NR1H4, IL1RL1, VIPR2, IL13RA2, CCL17, CCL22, HRH1, TH, COL6A1, CCL2, CD1A, HPGD, CFH, CYSLTR2, FCER2, CSF3R, ALOX15, SELE, PTGDR2, IL5RA, CCL23, IGHE, CD200R1, P2RX7, IL13, ADRA1D, GLIPR2, IL17RB, CCR4, NT5E, NLRP12, CCL26.	1.78 × 10^-09^ - 5.7 × 10^-03^
**PHYSIOLOGICAL SYSTEM DEVELOPMENT AND FUNCTION**	***P*-value**
**Haematological System Development and Function**	
GATA1, CRLF2, VIPR2, IL1RL1, SOCS2, IL13RA2, P2RX1, CCL17, CCL22, HRH1, NFIL3, TFF2, CCL2, CD1A, CFH, CYSLTR2, FCER2, CSF3R, ALOX15, PTGDR2, SELE, IL5RA, IGHE, CCL23, CD200R1, CD1B, ULBP1, P2RX7, IL13, ZBTB32, IL17RB, CCR4, NLRP12, NT5E, MYOCD, CCL26, CYP4A11.	4.79 × 10^-12^ - 6.71 × 10^-03^
**Tissue Morphology**	
GATA1, CRLF2, VIPR2, IL1RL1, NR1H4, IL13RA2, SLC9A4, CCL22, TH, HRH1, NFIL3,TFF2, CCL2, CFH, SMPD3, FCER2, CSF3R, ALOX15, SELE, PTGDR2, IL5RA, IGHE, CD200R1, P2RX, IL13, OXT, IL17RB, CCR4, NLRP12, NT5E, FLNC, CNN1, CYP4A11.	4.79 × 10^-12^ - 6.49 × 10^-03^
**Humoral Immune Response**	
HRH1,PTGDR2, NFIL3, IL5RA, IL17RB, CRLF2, CCR4, IGHE, IL1RL1, IL13RA2, IL13, HRH4, FCER2.	2.28 × 10^-11^ - 6.53 × 10^-03^
**Immune Cell Trafficking**	
IL1RL1, VIPR2, SOCS2, CCL17, CCL22, HRH1, NFIL3, TFF2, CCL2, CD1A, CFH, CYSLTR2, FCER2, CSF3R, ALOX15, PTGDR2, SELE, IGHE, CCL23, CD200R1, ULBP1, CD1B, P2RX7, IL13, IL17RB, CCR4, NT5E, NLRP12, CCL26.	5.04 × 10^-10^ - 6.71 × 10^-03^
**Cell-mediated Immune Response**	
PTGDR2, SELE, GATA1, CCR4, CCL2, IL1RL1, CCL23, IL13RA2, CCL17, CCL22, IL13.	1.09 × 10^-08^ - 5.63 × 10^-03^
**Lymphoid Tissue Structure and Development**	
CSF3R, ALOX15, SELE, GATA1, CRLF2, IL1RL1, IL13RA2, CCL17, CCL22, P2RX7, IL13, CCL2, CCR4, NT5E, CYSLTR2	3.82 × 10^-07^ - 5.7 × 10^-03^
**Digestive System Development and Function**	
TFF2, NR1H4, IL13RA2, SLC9A4, IL13	2.41 × 10^-05^ - 4.94 × 10^-03^
**b. S vs. C comparison**	
**DISEASES AND DISORDERS**	***P*-value**
**Hypersensitivity Response**	
FCER2, IGHE, SELE, VIPR2.	1.76 × 10^-06^ - 3.31 × 10^-02^
**Inflammatory Disease**	
FCER2, IGHE, IGKC, NT5E, SELE, VIPR2	4.64 × 10^-05^ - 4.10 × 10^-02^
**PHYSIOLOGICAL SYSTEM DEVELOPMENT AND FUNCTION**	***P*-value**
**Hematological System Development and Function**	
FCER2, IGHE, IGKC, SELE, NT5E, VIPR2.	1.76 × 10^-06^ - 4.57 × 10^-02^
**Immune Cell Trafficking**	
FCER2, IGHE, NT5E, SELE, VIPR2.	1.76 × 10^-06^ - 4.57 × 10^-02^
**c. R vs. S comparison**	
**DISEASES AND DISORDERS**	***P*-value**
**Gastrointestinal Disease**	
CCL26, IL13.	9.85 × 10^-06^ - 2.62 × 10^-02^
**Hematological Disease**	
CCL26, CD1A, IL13, FCER2, IGHE, IGKC, NT5E, SELE, VIPR2.	9.85 × 10^-06^ - 2.62 × 10^-02^
**PHYSIOLOGICAL SYSTEM DEVELOPMENT AND FUNCTION**	**P-value**
**Hematological System Development and Function**	
CCL26, CD1A, IL13.	2.35 × 10^-05^ - 4.76 × 10^-02^
**Immune Cell Trafficking**	
CCL26, CD1A, IL13.	2.35 × 10^-05^ - 4.76 × 10^-02^

The network Cell-To-Cell Signaling and Interaction, Hematological System Development and Function, Immune Cell Trafficking with a *P* score of 24 was the only network identified for the S vs. C dataset (Table 4b); and Antigen Presentation, Lipid Metabolism, Small Molecule Biochemistry with a *P* score of 18 was identified for the R vs. S comparison. Within these networks there were no Bio Functions beyond the 10^-10^ threshold (Table 4c).

The most significant network was Humoral Immune Response, Protein Synthesis, Inflammatory Response (R vs. C comparison), with *IL13* as the central gene (Figure 2). *IL13* was the top ranked gene in the R vs. C comparison, up-regulated 3.17 fold (*p* = 7.44 × 10^-7^) in resistant sheep. This was confirmed by the RT-qPCR analysis where it was 11.92 fold (*p* = 0.001) increased in the resistant group (Table 2). In this analysis it was also significantly up-regulated in susceptible animals in the S vs. C comparison (3.37 fold *p* = 0.03) and consequently 3.54 fold increased in resistant vs. susceptible sheep (*p* = 0.002).

### Mapping of sequencing tags to the sheep Oar v3.1 genome assembly

A previous study [24] also performed transcriptome analysis on the abomasal lymph nodes of these resistant and susceptible sheep using Illumina digital gene expression analysis. These data were originally analysed in relation to the *Bos taurus* genome assembly (Btau4.0). Here, we reanalysed the same primary data (ArrayExpress E-MTAB-445) against the most recent *Ovis aries* genome assembly (Oar v3.1) to obtain more accurate gene mapping. The mean total number of reads of the samples from 15 sheep (5 resistant, 5 susceptible and 5 control) was 1 473 000, of which 288 753 mapped to Btau4.0, with a maximum of 1 mismatch from an average tag length of 17 bases; in contrast 865 844 tags mapped the Oar v3.1. A ~2-4 fold increase in the number of significantly differentially expressed genes (Additional file 2) was also noted when comparing Oar v3.1 to Btau4.0; 229 in Oar v3.1 and 131 in Btau4.0 in the R vs. C comparison, 150 and 37 in the S vs. C comparison, and 146 and 83 in the R vs. S comparison.

IPA analysis of the revised digital gene expression data identified the top two networks (Additional file 3) in the R vs. C comparison as Cellular Growth and Proliferation, Cell Morphology, Cell-mediated Immune Response with a *P*-score of 56 and Post-Translational Modification, Hematological Disease, Cell Cycle, *P*-score of 52. The top network in the S vs. C comparison was Post-Translational Modification, Cell Signaling, DNA Replication, Recombination, and Repair with a P-score of 54. The top Bio Functions (Additional file 4) within these networks include Cancer, Gastrointestinal Disease, Dermatological Disease and Conditions and Immunological Disease; however, none of these Bio Functions reached the *p* ≤ 10^-10^ threshold.

## Discussion

This project is the logical extension of our three previous studies [24,32,33] concerned with the genetics and immunology of resistant and susceptible Blackface sheep persistently infected with the common abomasal nematode parasite, *T. circumcincta*. The first [32] described host-parasite interactions in a single intensively-phenotyped cohort with variable susceptibility; which were exploited to help analyse the nature of the mature host response associated with differential resistance [24,33]; although this misses early events associated with the development of immune responses The aim of the current study was to identify genes and physiological pathways associated with the differential activation of the immune response, linked to the maintenance of resistance and susceptibility. It is part of a larger project that eventually aims to identify candidate genes that could be used as selectable markers of resistance in these Blackface sheep as well as other commercial sheep breeds.

The study exploits a new genomics resource for the analysis of the sheep transcriptome; the homologous Affymetrix Gene 1.1 ST Array based on the *Ovis aries* Oar v2 genome assembly. This consists of 508 538, 25mer probes for 22 047 genes, which interrogate approximately 625 bases per gene covering all exons of each transcript. Also new to this study is the use and validation of the SABiosciences Cow Inflammatory Cytokines & Receptors RT^2^ Profiler™ PCR Array in sheep; enabling 83 RT-qPCR assays to be used to validate the arrays rather than the more usual 8 or 10 genes. In addition we reanalysed first generation Illumina digital gene expression data [24] using the latest sheep genome assembly (Oar v3.1) with significantly different results; more than 3 times the number of tags mapped to Oar v3.1 as originally mapped to Btau 4.0. The only other study [40] that has examined the sheep ALN transcriptome used a small array of 1480 annotated probes with 5373 unannotated expressed sequence tags. This identified only one differentially expressed gene (*HSPA1A*) in relation to *H. contortus* infection.

The sheep in this study that were predicted to show variation in resistance to *T. circumcincta* were trickle-infected regularly for 3 months to mimic natural infection and continual exposure. They were analysed when the mature immune response of the resistant animals had controlled and/or eliminated that infection. At the same time susceptible animals did not control infection, and retained adult nematodes that produced large numbers of eggs [32]. A previous study showed that these susceptible sheep generated an active Th17 immune and inflammatory response that failed to control infection [33]. In this current study the results of the array analysis focused on the nature of the mature host response in the resistant animals, which controls nematode colonization despite chronic exposure to infectious larvae.

Murine models of nematode parasite immunity, resistance and susceptibility, using the nematodes *Heligmosomoides polygyrus*, *Nippostrongylus brasiliensis* and *Trichuris muris* are linked to strong Th2 responses, indicated by high levels of the cytokines interleukin 4 (IL4) and IL13 with parasite immunity and resistance, and high levels of interferon γ (IFNγ) with susceptibility [41-43]. It is clear from the IPA in this study that the host response linked to parasite control is strongly associated with the network “Humoral Immune Response, Protein Synthesis, Inflammatory Response” and that many of the network genes are key regulators of a Th2 cell response and are strongly up-regulated in the resistant sheep. This is in agreement with the results from the array analysis of the mucosa from challenge-treated-reinfected “immune” sheep [29]. IL4 and IL13 are the two cytokines that principally control Th2 differentiation [44,45]; both were significantly increased in resistant sheep, *IL4* (3.96 fold in R vs. C) in the RT-qPCR and *IL13* in both array (4.76 fold in R vs. C and 3.17 fold in R vs. S) and RT-qPCR (11.92 fold in R vs. C and 3.54 in S vs. C). These cytokines have related receptors and *IL13RA2* is also up-regulated in resistant sheep; binding to their cognate receptors stimulates the activation of STAT6 that controls the expression of GATA3, the prime transcription factor for Th2 differentiation [46]. IL5 is the third archetypal Th2 cytokine, and is also increased in resistant sheep (3.4 fold in R vs. S by RT-qPCR). A major function of IL5 is in stimulating eosinophil maturation and localization; these are crucial effector cells in the exclusion of *T. circumcincta* through binding to parasite-specific IgG, IgE and IgA antibodies and subsequent degranulation [13]. Eosinophils are most numerous in the mucosa of the resistant sheep [47] evidenced by the increased expression, by array, of both *FCER2* (1.72 fold in R vs. S) the low affinity receptor of IgE, and the IL5 receptor (*IL5RA* 3.15 fold in R vs. C) both expressed by eosinophils (Table 1).

Sheep control larval colonization, worm development and egg production through the generation of parasite-specific IgA and IgE antibodies [7,9,11,20]. Levels of these two antibody classes are highly negatively correlated with worm length and fecundity and FEC [32] and both IgA (*IGHA* 2.01 fold) and IgE (*IGHE* 17.21 fold) are significantly increased (both by array) in the resistant sheep. The generation of Th2 responses in the gastrointestinal tract seem to be induced by the events at the mucosal epithelial surface [48]. The intestinal epithelium is activated by parasite antigens via innate receptors and secrete the cytokines IL25 and IL33 that act on the newly identified innate lymphoid cells (ILC2) [49], and TSLP that acts on dendritic cells (DC) [50]. This leads to IL13 expression by ILC2 and Th2 activation by the DCs. Major parts of the receptors for these cytokines are up-regulated (by array) in resistant sheep; *IL17RB* (receptor for IL25) is increased 4.73 fold, *IL1RL1* (IL33 receptor and the principal marker for ILC2) in increased 2.7 fold and *CRLF2* (TSLP receptor) is increased 2.03 fold, in the R vs. C comparison. The endothelia and DCs in the abomasal lymph nodes of resistant sheep express significantly increased levels of *HRH1* (1.57 in R vs. C). Histamine receptor positive DCs modulate Th1/Th2 balance by inhibiting IL12R1 signalling thus promoting Th2 responses [51].

However, resistance and susceptibility is not just a matter of Th1/Th2 discrimination as susceptible sheep also show increased expression of *IL4* (3.83 fold in S vs. C) and *IL13* (3.37 fold in S vs. C) by RT-qPCR and *IGHE* (13.25 fold in S vs. C) by array. *IGHA* is only marginally increased (but non-significant) in these susceptible animals. Nevertheless the mature tissue response of the resistant and susceptible sheep is distinct [33]. The abomasal mucosa of resistant sheep, at least 6 weeks after the last positive FEC, has evidence of only minor pathological change with small numbers of infiltrating lymphocytes and eosinophils. In contrast the mucosa of susceptible sheep was grossly inflamed.

These pathological differences are reflected in the differential expression of the chemokines, molecules which control leukocyte movement. The high expression of *CCL17* (3.33 fold in R vs. C in array) and *CCL26* or eotaxin-3 (15.5 fold in R vs. C in array) begins to explain the nature of mucosal infiltrate of resistant sheep, which consists largely of lymphocytes and eosinophils [52,53]. Indeed CCL17 is chemotactic for, and activates, CCR4+ Th2 cells; their expression of IL4 and IL13 stimulates the expression of *CCL26*, which is chemotactic for eosinophils and basophils. IL13 also stimulates the expression of CCL5 (*CCL5* 3.15 fold in R vs. C in RT-qPCR) by activated T and NK cells, which is chemotactic for memory T cells and promotes Th2 responses [54].

Changes to these and many of the other genes indicate that a principal component of the response of resistant animals also includes a repression of acute inflammation and tissue healing. The low levels of *MIF* (−2.16 in R vs. C in array) and *SELE* (−1.8 in R vs. C in array) begins to explain the paucity of neutrophils in resistant animals [55]. A principal function of MIF is induction of pro-inflammatory cytokines, which stimulates *SELE* (E-selectin) expression and hence neutrophil localization [56]; however the level of *SELE* in resistant sheep is significantly repressed, possibly explaining the lack of neutrophils in the abomasal mucosa of these animals [33]. Three other genes which play significant roles in the regulation of inflammation are increased in resistant sheep. *ALOX15* (lipoxygenase 15) is induced by IL4 and IL13 and inhibits pro-inflammatory leukotrienes and suppresses neutrophil chemotaxis; *NR1H4* plays an key role in cholesterol homeostasis and inhibits IL1β induced inflammation [57]; *CD200R* is the Ox-2 receptor expressed on myeloid cells and functions to down-regulate myeloid cell activation and therefore depress inflammation [58]. Both resistant and susceptible sheep are equally affected for the first 6 weeks of trickle infection [32] but only resistant animals control infection. Consequently the abomasum of those animals begins to heal; and this is seen by the significant changes (R vs. S and R vs. C) to genes associated with healing, with an 8 – 10 fold increase in type VI collagen (*COL6A5*) and a 3 fold increase in enteric smooth muscle actin (*ACTG2*).

*VIPR2* (2.04 in S vs. C) is the one gene in the array that is differentially-expressed in susceptible sheep but not in the resistant group. This is the vasoactive intestinal peptide receptor; ligand-receptor interactions leads to inhibition of IL2-driven T cell proliferation and chemoattraction [59], consistent with the immunopathology of the susceptible abomasum. The excretory/secretory antigens of *T. circumcincta* have been shown to induce the expression of FoxP3 by murine T cells in vitro [60]; however there is no evidence for any increase in Tregs in the parasite-free resistant or highly-infected susceptible sheep.

The ultimate aim of these studies was to identify candidate genes as potential selectable markers for resistance to *T. circumcincta* infection. Within this study we have highlighted a small number of physiological pathways associated with differential susceptibility in Blackface sheep; in particular identified genes linked to differential T cell polarization. The Affymetrix ovine gene array incorporates probes for all the annotated exons within the sheep genome and a major advantage of using this technology is that differential transcript usage can be identified directly. We are currently analysing the array data to identify transcript variants of genes within the top-networks that are differentially-expressed in relation to resistance and susceptibility.

## Competing interests

The authors declare that they have no competing interests.

## Authors’ contributions

JH conceived the study, collected the samples and is the principal investigator. AGG and JH designed the current experiments; AGG performed the Affymetrix and RT-qPCR experiments and was responsible for the statistical, bioinformatic and pathway analysis. DB performed the original digital gene expression study, including the bioinformatics. HW and AJ reanalyzed the digital gene expression in relation to Oar v3.1. JH drafted the manuscript. All the authors have read and approved the final manuscript.

## Supplementary Material

Additional file 1 **Digital gene expression analysis of gastric lymph node of *T. circumcincta *infected sheep.** Differentially expressed genes in the R vs. C, S vs. C and R vs. S comparisons analysed using the *Ovis aries* Oar v3.1 genome assembly. Mean; ΔΔCt (mean Ct^GOI^ - meanC^*YWHAZ*^). Click here for file

Additional file 2 **RT-qPCR analysis of gastric lymph node of *T. circumcincta *infected sheep.** Differentially expressed genes in the R vs. C and S vs. C comparisons. Mean R, S, C; mean tag numbers per 10^6^ tags in Resistant (R), Susceptible (S), Control (C). FC R/C, S/C, R/S; fold change Resistant vs. Control (R/C), Susceptible vs. Control (S/C, Resistant vs. Susceptible (R/S). Click here for file

Additional file 3 ***Top networks identified by Ingenuity Pathway Analysis, from the Illumina digital gene expression data.*** Network *P*-scores [−log10 (*P*-value)] is the probability of a network being randomly generated. Click here for file

Additional file 4 **Top Bio Functions identified by Ingenuity Pathway Analysis, from Illumina digital gene expression data. ***P*-values calculated by Fisher’s exact test. Click here for file
