# Supplementary material for: Exploring the abomasal lymph node transcriptome for genes associated with resistance to the sheep nematode Teladorsagia circumcincta
Source: Vet Res. 2013 Aug 8;44(1):68. doi: 10.1186/1297-9716-44-68 (PMC3751673; doi:10.1186/1297-9716-44-68)
Supplement: Additional file 3 — Top networks identified by Ingenuity Pathway Analysis, from the Illumina digital gene expression data. Network P-scores [−log10 (P-value)] is the probability of a network being randomly generated. [file 1297-9716-44-68-S3.docx]

**Additional File 3 Top networks identified by Ingenuity Pathway Analysis, from the Illumina Solexa digital gene expression data.**

| Resistant vs. Control | Score | |
| --- | --- | --- |
| Cellular Growth and Proliferation, Cell Morphology, Cell-mediated Immune Response  Post-Translational Modification, Hematological Disease, Cell Cycle  Infectious Disease, Neurological Disease, Antimicrobial Response  Developmental Disorder, Endocrine System Disorders, Gastrointestinal Disease  Post-Translational Modification, Developmental Disorder, Hereditary Disorder | | 56  52  28  28  28 |
| Susceptible vs. Control | | |
| Post-Translational Modification, Cell Signaling, DNA Replication, Recombination, and Repair  Developmental Disorder, Hereditary Disorder, Metabolic Disease  Cell Morphology, Cellular Assembly and Organization, Cellular Function and Maintenance  Molecular Transport, Protein Trafficking, Post-Translational Modification  Cell Morphology, Hematological System Development and Function, Hematopoiesis | | 54  32  28  28  24 |
| Resistant vs. Susceptible | | |
| Cellular Assembly and Organization, Cellular Function and Maintenance, Cellular Movement  Cellular Development, Cellular Growth and Proliferation, Hematological System Development and Function  Carbohydrate Metabolism, Connective Tissue Disorders, Developmental Disorder  Hereditary Disorder, Metabolic Disease, Cardiovascular Disease  Hereditary Disorder, Neurological Disease, Cell Death and Survival | | 35  33  29  29  27 |
