# Supplementary material for: Exploring the abomasal lymph node transcriptome for genes associated with resistance to the sheep nematode Teladorsagia circumcincta
Source: Vet Res. 2013 Aug 8;44(1):68. doi: 10.1186/1297-9716-44-68 (PMC3751673; doi:10.1186/1297-9716-44-68)
Supplement: Additional file 4 — Top Bio Functions identified by Ingenuity Pathway Analysis, from Illumina digital gene expression data. P-values calculated by Fisher’s exact test. [file 1297-9716-44-68-S4.docx]

**Additional File 4 Top Bio Functions identified by Ingenuity Pathway Analysis, from Illumina digital gene expression data.**

**A. R vs. C comparison**

| **DISEASES AND DISORDERS** | ***P*-value** |
| --- | --- |
| **Cancer** |  |
| DBN1, ASPSCR1, MYL6, RBMX2, NOMO1 (includes others), ASCC2, SIGLEC10, EIF2A, RPS11, LAMC1, MGAT4B, ZUFSP, CBX8, TIMM44, RPL3, VRK2, RPA1, TMX2, CCND2, ICAM3, PSMB1, ZYX, ALDOA, U2AF1, TBC1D5, UBE2C, HSD17B10, PBX2, ARHGEF7, IGLL1/IGLL5, ALDH1L1, SURF4, SMURF1, PRCC, SSR2, PSMB9, MX2, TNC, N4BP2L2, PPIF, EMP3, HMHA1, CBX4, TNNI1, CSTF3, AKAP1, PINK1, LIG1, TNFRSF4, DNMT3A, VDAC2, CTBP1, USP7, TNIP1, CNDP2, PRMT1, UBA7, PSMA6, EWSR1, C3, HDAC2, IER2, WEE1, SP140, GRIP1, RANBP1, ERCC2, KRBA1, TBL1X, SSRP1, NCOA3, PYGM, SCRIB, RBP4, INO80B, CARS, MRPL45, USP11, SAT1, SLC15A3, CLEC3B, PDCD4, DNAH8, ANGPTL4, RPS16, EIF2S3, PPP2R5C, HCST, VASP, RPL29, EMILIN1, TACC3, FAM134B, TPM2, IL1R1, MANBA, GCN1L1, UBE3A, STT3A, TBL2, ENO1, NDUFV2, LGALS3BP, HMGCR. | 1.19 × 10^-04^ - 4.81 × 10^-02^ |
| **Gastrointestinal Disease** |  |
| LIG1, TACC3, TNC, C3, VRK2, IGLL1/IGLL5, IL1R1, ADAR, ERCC2, TNIP1, PRCC, ANGPTL4, RPS16, SCRIB, HCST, AKAP1, UBE2C. | 1.19 × 10^-04^ - 4.46 × 10^-02^ |
| **Dermatological Diseases and Conditions** |  |
| LIG1, DBN1, SAT1, CLEC3B, CTBP1, AQR, TNIP1, IFI6, EMILIN1, PSMA6, TNC, C3, RPL3, PPIF, IER2, PSMD6, ALOX5AP, GRIP1, RANBP1, MANBA, IL1R1, ERCC2, ADAR, KPNB1, CCND2, LGALS3BP, OTUB1, ZYX, PSAP, HMGCR, POLR2I, PCBD1, DOCK10, UBE2C, RBP4. | 1.46 × 10^-04^ - 4.15 × 10^-02^ |
| **Reproductive System Disease** |  |
| LIG1, HSD17B10, PSMB9, PSMA6, TNC, PPIF, HDAC2, MYL6, EMP3, WEE1, TPM2, MANBA, CLEC3B, ERCC2, UBE3A, TBL1X, ENO1, TNIP1, NDUFV2, ALDOA, ZYX, EMILIN1, UBE2C. | 3.47 × 10^-04^ - 3.13 × 10^-02^ |
| **Infectious Disease** |  |
| TNFRSF4, DNMT3A, SMURF1, CTBP1, AQR, UBA7, RPS16, IFI6, MX2, C3, HDAC2, RPL3, ALOX5AP, PSMD6, IL1R1, MANBA, ADAR, SSRP1, NCOA3, STAT4, KPNB1, ZYX, HMGCR, POLR2I, HIST1H2BN, DOCK10, UBE2C. | 6.53 × 10^-04^ - 4.86 × 10^-02^ |
| **PHYSIOLOGICAL SYSTEM DEVELOPMENT AND FUNCTION** | ***P*-value** |
| **Cell Death and Survival** |  |
| LIG1, TNFRSF4, DNMT3A, EIF2A, VDAC2, AFAP1L2, CTBP1, USP7, TNIP1, UBA7, PACS2, PSMA6, EWSR1, C3, HSH2D, HDAC2, WEE1, PSMD6, RPA1, RANBP1, ERCC2, ADAR, SSRP1, NCOA3, ASAH1, NFKBID, CCND2, ICAM3, TTF1, SCRIB, PSMB1, ZYX, ALDOA, PKMYT1, GNAL, UBE2C, RBP4, HSD17B10, CARS, ARHGEF7, USP11, SAT1, VTI1B, PDCD4, SMURF1, MCOLN1, ANGPTL4, PPP2R5C, IFI6, HCST, NSMCE1, VASP, PTPN7, TACC3, TNC, PPIF, EMP3, FAM134B, ATG3, IL1R1, UBE3A, PPM1G, STAT4, ENO1, NDUFV2, LGALS3BP, BFAR, PSAP, CBX4, HMGCR, AKAP1, PINK1. | 7.45 × 10^-06^ - 4.97 × 10^-02^ |
| **Cellular Development** |  |
| EWSR1, TNFRSF4, C3, ARHGEF7, IGLL1/IGLL5, IL4I1, TPM2, SIGLEC10, IL1R1, UBE3A, NCOA3, STAT4, LAMC1, PRMT1, CCND2, VASP. | 3.04 × 10^-04^ - 4.46 × 10^-02^ |
| **Cellular Growth and Proliferation** |  |
| LIG1, DBN1, TNFRSF4, SIGLEC10, AFAP1L2, GGA2, LAMC1, CTBP1, USP7, PRMT1, MGAT4B, HSH2D, EWSR1, C3, METTL3, APPL2, HDAC2, WEE1, IL4I1, RPA1, ADAR, ERCC2, ASAH1, NCOA3, NFKBID, CCND2, PYGM, ICAM3, TTF1, SCRIB, ZYX, INO80B, UBE2C, RBP4, IGLL1/IGLL5, SAT1, ALDH1L1, VTI1B, SURF4, PDCD4, HADHB, ANGPTL4, PRCC, PPP2R5C, VASP, RPL29, EMILIN1, TACC3, TNC, EMP3, TPM2, CINP, IL1R1, UBE3A, PPM1G, STAT4, ENO1, PSAP, CBX4, HMGCR. | 3.04 × 10^-04^ - 4.46 × 10^-02^ |
| **Gene Expression** |  |
| RPL24, PBX2, POLR2F, EIF2B4, TNFRSF4, DNMT3A, MRPL2, EIF2A, AFAP1L2, PDCD4, CTBP1, TNIP1, TCF25, EIF2S3, PPP2R5C, RPL29, VASP, CBX8, PTPN7, EWSR1, TNC, HDAC2, N4BP2L2, VRK2, SP140, RPA1, GRIP1, ERCC2, TBL1X, SSRP1, UBE3A, NCOA3, PHRF1, PRMT7, STAT4, NFKBID, ENO1, OTUB1, TTF1, ELP4, SUPT3H, CBX4, TNNI1, POLR2I, PCBD1. | 5.32 × 10^-04^ - 3.13 × 10^-02^ |
| **DNA Replication, Recombination, and Repair** |  |
| LIG1, HDAC2, DNMT3A, WEE1, OTUB1, TTF1, RPA1, U2AF1, ERCC2, ADAR, SSRP1, ACTL6A. | 1.08 × 10^-03^ - 4.15 × 10^-02^ |

**B. S vs. C comparison**

| **DISEASES AND DISORDERS** | ***P*-value** |
| --- | --- |
| **Dermatological Diseases and Conditions** |  |
| LIG1, BLNK, SNRPE, EXOSC10, RBBP7, SAT1, MED28, CLEC3B, CTLA4, CTBP1, TNIP1, TGFB1, KEAP1, FABP4, IFI6, EMILIN1, TNC, C3, DDX23, ALOX5AP, RPA1, MANBA, IL1R1, JUNB, XAB2, CCND2, BSG, H2AFY, RECQL4, LGALS3BP, FSCN1, OTUB1, ZYX, RPS6KA4, GSTP1, UBE2C, RBP4. | 3.42 × 10^-05^ - 1.57 × 10^-02^ |
| **Cardiovascular Disease** |  |
| PFN1, C3, TGFB1, JUNB, IL1R1, CTLA4, GSTP1. | 4.68 × 10^-04^ - 1.42 × 10^-02^ |
| **Inflammatory Disease** |  |
| USP7, NFKBID, C3, BSG, TGFB1, CORO1A, RPS6KA4, JUNB, IL27RA, IL1R1, CTLA4, GSTP1. | 4.68 × 10^-04^ - 1.57 × 10^-02^ |
| **Cancer** |  |
| LIG1, ASPSCR1, PFN1, SNRPE, RBBP7, SAT1, SMC4, MED28, CLEC3B, PPP4C, CTLA4, SPDEF, SCNN1A, USP7, TNIP1, TGFB1, CSNK2A1, EIF3A, FABP4, IL27RA, DBF4, EMILIN1, CBX8, TACC3, C3, TNC, PAM, TIMM44, GRB2, HMHA1, STOML2, RPA1, JUNB, MRPS14, MANBA, CCND2, BSG, NDUFV2, H2AFY, RECQL4, FSCN1, LGALS3BP, ZYX, ALDOA, CBX4, GSTP1, PINK1, RBP4, UBE2C. | 7.37 × 10^-04^ - 1.87 × 10^-02^ |
| **Inflammatory Response** |  |
| C3, PFN1, GRB2, ALOX5AP, IL1R1, JUNB, CTLA4, SPDEF, NFKBID, TGFB1, CORO1A, FABP4, IGHA1, IL27RA, GSTP1. | 9.03 × 10^-04^ - 1.77 × 10^-02^ |
| **PHYSIOLOGICAL SYSTEM DEVELOPMENT AND FUNCTION** | ***P*-value** |
| **Haematological System Development and Function** |  |
| LIG1, BLNK, TACC3, TNC, C3, GRB2, ALOX5AP, RPA1, JUNB, IL1R1, CTLA4, EEF1D, NFKBID, CCND2, BSG, TGFB1, CORO1A, CSNK2A1, FABP4, RPS6KA4, IGHA1, IL27RA, CD84, GSTP1. | 5.01 × 10^-05^ - 1.77 × 10^-02^ |
| **Hematopoiesis** |  |
| BLNK, TACC3, GRB2, RPA1, JUNB, IL1R1, CTLA4, EEF1D, NFKBID, CCND2, BSG, TGFB1, CSNK2A1, IL27RA, GSTP1. | 5.01 × 10^-05^ - 1.52 × 10^-02^ |
| **Lymphoid Tissue Structure and Development** |  |
| BLNK, LIG1, TACC3, C3, GRB2, IL1R1, JUNB, CTLA4, EEF1D, NFKBID, CCND2, BSG, TGFB1, RECQL4, CSNK2A1, IL27RA, CD84. | 5.01 × 10^-05^ - 1.79 × 10^-02^ |
| **Tissue Morphology** |  |
| LIG1, BLNK, TACC3, C3, SAT1, IL1R1, JUNB, CTLA4, SPDEF, EEF1D, LAMC1, MCOLN1, CCND2, TGFB1, CORO1A, CSNK2A1, IL27RA, CD84, GSTP1. | 5.01 × 10^-05^ - 1.64 × 10^-02^ |
| **Connective Tissue Development and Function** |  |
| LIG1, C3, GRB2, SAT1, JUNB, EEF1D, CTBP1, LAMC1, BSG, TGFB1, RECQL4, CSNK2A1, FABP4, CBX4, UBE2C. | 2.94 × 10^-04^ - 1.42 × 10^-02^ |

**C. R vs. S comparison**

| **DISEASES AND DISORDERS** | ***P*-value** |
| --- | --- |
| **Gastrointestinal Disease** |  |
| TLR10, ANGPTL4, CXCL12, CCL21, HCST, ATR. | 1.37 × 10^-04^ - 1.34 × 10^-02^ |
| **Immunological Disease** |  |
| RNF139, CFD, CD19, ARFGAP3, TNFRSF4, DNASE1L3, CXCL12, ALOX5AP, NFKB1, GSN, PHRF1, ENO1, LAT, CCL21, PHF1, HMGCR. | 1.37 × 10^-04^ - 2.69 × 10^-02^ |
| **Inflammatory Disease** |  |
| RNF139, CFD, CD19, ARFGAP3, DNASE1L3, CXCL12, GSN, NFKB1, PHRF1, ENO1, TLR10, CCL21, PHF1, HMGCR. | 1.37 × 10^-04^ - 2.69 × 10^-02^ |
| **Inflammatory Response** |  |
| PSMB9, CD19, TNFRSF4, CXCL12, RAC1, ALOX5AP, NFKB1, GSN, VTI1B, ANGPTL4, LAT, CCL21, HMGCR. | 1.37 × 10^-04^ - 2.89 × 10^-02^ |
| **Ophthalmic Disease** |  |
| POLG2, TMEM126A, CXCL12, CCL21, GRIP1. | 1.37 × 10^-04^ - 2.69 × 10^-02^ |
| **PHYSIOLOGICAL SYSTEM DEVELOPMENT AND FUNCTI** | ***P*-value** |
| **Cell-mediated Immune Response** |  |
| TNFRSF4, LAT, CXCL12, CCL21, RAC1. | 1.37 × 10^-04^ - 3.31 × 10^-02^ |
| **Haematological System Development and Function** |  |
| PSMB9, CD19, TNFRSF4, ATG3, RAC1, CXCL12, ALOX5AP, SIGLEC10, GSN, NFKB1, CBX5, ANGPTL4, ICAM3, LAT, CCL21, HMGCR, HCST, CD84, BST1. | 1.37 × 10^-04^ - 3.31 × 10^-02^ |
| **Hematopoiesis** |  |
| CD19, TNFRSF4, CCL21, CXCL12, RAC1, NFKB1, BST1. | 1.37 × 10^-04^ - 2.69 × 10^-02^ |
| **Immune Cell Trafficking** |  |
| CD19, TNFRSF4, LAT, RAC1, CXCL12, CCL21, ALOX5AP, GSN, NFKB1. | 1.37 × 10^-04^ - 3.31 × 10^-02^ |
| **Humoral Immune Response** |  |
| CD19, CCL21, CXCL12, RAC1, SIGLEC10, CD84, NFKB1, BST1. | 1.99 × 10^-03^ - 2.69 × 10^-02^ |
